# Metabolic Dysregulation and Its Multifaceted Impact on Cardiovascular Autonomic Control in Type 2 Diabetes Mellitus: Insights From Comprehensive Assessment

**DOI:** 10.7759/cureus.59776

**Published:** 2024-05-07

**Authors:** Amrutha Kanagala, Harsoda J M

**Affiliations:** 1 Physiology, Smt. B. K. Shah Medical Institute & Research Centre, Sumandeep Vidyapeeth Deemed to be University, Vadodara, IND

**Keywords:** cardiovascular autonomic function, inflammatory markers, duration of diabetes, bmi, lipid profile, glycemic variability, hba1c, type 2 diabetes mellitus

## Abstract

Background: Type 2 diabetes mellitus (T2DM) is associated with a spectrum of metabolic dysfunctions that significantly elevate the risk of cardiovascular disorders. Understanding the intricate relationship between metabolic control and cardiovascular autonomic function in individuals with T2DM is crucial for effective management and the prevention of associated complications. This insight is foundational in developing targeted strategies that can mitigate the heightened cardiovascular risks inherent to this condition, thereby enhancing patient outcomes and quality of life.

Aim: The primary aim of the study was to explore the interconnections between various aspects of metabolic control in individuals with T2DM. This includes examining how glycemic variability, lipid profiles, body mass index (BMI), duration of diabetes, inflammatory markers, and cardiovascular autonomic function are interrelated.

Methods: A cross-sectional study was conducted involving 100 individuals with T2DM and 100 control participants. HbA1C levels, glycemic variability, lipid profile, BMI, duration of diabetes, and inflammatory markers were assessed. Cardiovascular autonomic function parameters, including resting heart rate and blood pressure responses, were evaluated using standardized tests.

Results: People with T2DM had significantly higher levels of glycosylated hemoglobin (HbA1C) compared to controls (mean difference = 2.95%, p < 0.001). Elevated HbA1C levels were correlated with increased resting heart rate (mean difference = 10 bpm, p < 0.001) and aberrant blood pressure responses during autonomic function assessments (p < 0.01 for systolic blood pressure; p < 0.05 for diastolic blood pressure). Glycemic variability (correlation coefficient (𝑟) = 0.75, p < 0.001) and dyslipidemia (elevated triglycerides and LDL cholesterol, reduced HDL cholesterol) were associated with cardiovascular autonomic dysfunction. Higher BMI values in T2DM individuals were independently correlated with alterations in autonomic function (𝑟 = 0.60, p < 0.001). The prolonged duration of diabetes was linked to greater impairment in autonomic function (mean decrease = 0.5 points per year, p < 0.01). In the T2DM group, higher levels of inflammatory markers (C-reactive protein and interleukin-6) were seen, which may have led to problems with the autonomic nervous system.

Conclusion: Metabolic dysregulation, such as high HbA1C levels, glycemic variability, dyslipidemia, obesity, having diabetes for a long time, and inflammation, is linked to cardiovascular autonomic dysfunction in T2DM. Early intervention targeting these metabolic abnormalities may mitigate the risk of cardiovascular complications in individuals with T2DM.

## Introduction

A decreased response to insulin and insufficient insulin production are two characteristics of diabetes mellitus type 2, also known as T2DM, a metabolic condition that lasts for a long time. This results in elevated levels of glucose in the blood and a disturbance in the mechanism by which the body regulates glucose metabolism [1,2]. It is a huge global public health problem, with an increasing occurrence and severe morbidity and fatality rates. People with T2DM have a higher likelihood of having different types of complications affecting the larger blood vessels (macrovascular) and smaller blood vessels (microvascular). Among these repercussions are complications such as cardiovascular illness, injury to the nerves (nerve damage), damage to the renal system (nephropathy), and impairment to the eyes (retinopathy) [3,4].

In patients with T2DM, cardiovascular disease is the leading cause of death and illness. It is also the cause of a significant number of deaths that are attributed to diabetes. Autonomic dysfunction, which shows up as problems controlling heart rate (HR), blood pressure (BP), and vascular tone [5,6], is a major cause of the higher risk of heart disease in people with T2DM. Autonomic dysfunction in diabetes is multifactorial, involving complex interactions between metabolic, neural, and vascular pathways [7,8].

Metabolic regulation is essential in the development of cardiovascular autonomic dysfunction in T2DM. People with diabetes are more likely to get heart disease and die if they have higher levels of hemoglobin A1C, also known as glycosylated hemoglobin (HbA1C), a marker of how well their blood sugar is controlled over time [9,10]. Furthermore, glycemic variability, which refers to the variations in levels of glucose in the blood, has been identified as a separate factor that can predict cardiovascular problems in individuals with T2DM [11].

Aside from the management of glycemic levels, dyslipidemia, obesity, the length of time a person has had diabetes, and inflammation are all significant metabolic abnormalities that lead to the development of cardiovascular autonomic dysfunction in people who have T2DM [12]. The development of atherosclerosis and the dysfunctional operation of the endothelium are both results of the condition known as dyslipidemia, which is characterized by elevated triglycerides, LDL cholesterol, and lower levels of HDL cholesterol. There is a correlation between this condition and an increased risk of cardiovascular disease in people who have T2DM [13]. A higher body mass index (BMI) indicates obesity, which is associated with insulin resistance (IR), abnormal blood lipid levels, and generalized body inflammation. All these factors contribute to problems with the autonomic nervous system and cardiovascular issues in T2DM [14].

Moreover, the length of time a person has had diabetes is a crucial factor in determining their risk of cardiovascular problems. Those who have had the condition for a longer period of time are more likely to have a higher occurrence and greater severity of autonomic dysfunction [15]. The pathophysiology of diabetes and cardiovascular disease has been linked to chronic low-grade inflammation, which is characterized by elevated levels of inflammatory markers like C-reactive protein (CRP) and interleukin-6 (IL-6). This highlights the complex interactions between metabolic dysregulation and cardiovascular autonomic dysfunction in T2DM [16].

Given the considerable impact that metabolic abnormalities have on cardiovascular autonomic function in T2DM, it is imperative to thoroughly understand the underlying biological mechanisms and pinpoint potential therapeutic targets. This deeper understanding is critical for the prevention and effective management of cardiovascular complications among this high-risk population. The study is designed to delve into the complex relationship between metabolic control and cardiovascular autonomic function in individuals diagnosed with T2DM. By doing so, it aims to provide comprehensive insights into the nuanced pathophysiology underlying diabetic cardiovascular complications. Additionally, the findings are expected to aid in the formulation of enhanced strategies for precise risk stratification and the development of targeted therapeutic interventions, ultimately improving patient care and outcomes in this vulnerable group.

## Materials and methods

Study setting

The study took place at the Department of Physiology within Mamata Medical College, which is based in Khammam, Telangana, India. Mamata Medical College serves as a critical tertiary care institution, delivering a range of healthcare services to a diverse demographic throughout the region. The Institutional Ethics Committee (IEC), noted under IRB/IEC-94, gave its approval for this particular study. This approval included a revision of the patient consent procedures to ensure alignment with the latest ethical standards and regulations. This modification was instrumental in safeguarding participant rights and maintaining the integrity of the research process. 

Study design

This study was done over a duration of 24 months, spanning from January 2022 to December 2023. The study's design was to evaluate the correlation between metabolic management and cardiovascular autonomic function in people diagnosed with T2DM in comparison to control participants.

Participant recruitment

The research project enlisted a total of two hundred volunteers, one hundred of whom were diagnosed with T2DM and one hundred of whom were control subjects who did not have diabetes. The participants were matched in terms of age and gender. Persons who had been diagnosed with T2DM were recruited from the outpatient department of Mamata Medical College. On the other hand, people who did not have T2DM were picked from the community through advertisements and referrals.

Inclusion criteria

Those who are recognized to have diabetes are those who have been diagnosed with T2DM and who are at least 18 years old were included in the study. Control volunteers did not have diabetes and were of the same age and gender. Participants who were willing to give informed consent to participate in the study were included.

Exclusion criteria

The exclusion criteria for the study were rigorously defined to maintain the integrity of the data collected. Individuals diagnosed with Type 1 diabetes mellitus or other forms of diabetes distinct from Type 2 were excluded to ensure a homogeneous study population specific to T2DM. Additionally, participants with a history of cardiovascular diseases, kidney issues, or other significant underlying health conditions were excluded to avoid confounding variables that could impact the study’s outcomes on cardiovascular autonomic function. The study also excluded individuals with medical conditions known to affect autonomic function, such as neuropathy or autonomic dysfunction. Lastly, women who were pregnant or currently breastfeeding were not eligible to participate, as these conditions could introduce physiological changes that might skew the results relevant to the diabetic population under study

Data collection

Each participant's age, gender, length of diabetes (for the T2DM group), and medical history were taken as baseline demographic and clinical data. Standardized methodologies were used to record anthropometric measures, which included height, weight, and BMI.

Laboratory investigations

Following an overnight fast, blood samples were obtained from each participant in order to determine their HbA1C levels, lipid profile (which included triglycerides (TGs), low-density lipoprotein (LDL) cholesterol, and high-density lipoprotein (HDL) cholesterol), and inflammatory markers (which included CRP and IL-6). Continuous glucose monitoring (CGM) devices were utilized in order to evaluate the effects of glycemic fluctuation.

Assessment of cardiovascular autonomic function

Cardiovascular autonomic function was evaluated using standardized tests, including HR variability analysis, deep breathing test, Valsalva maneuver, and orthostatic BP measurement. The results of these tests provide quantitative measurements of the function of the autonomic nervous system, which includes both sympathetic and parasympathetic activity levels.

Ethical considerations

The Mamata Medical College's Institutional Ethics Committee in Khammam, Telangana, India, authorized the study procedure. Informed consent was received from each and every participant prior to their registration in the study, and the confidentiality of their personal information was maintained throughout the duration of the study.

Statistical analysis

The required software, such as IBM SPSS Statistics for Windows, Version 25 (Released 2017; IBM Corp., Armonk, New York, United States) and R (R Foundation for Statistical Computing, Vienna, Austria), was utilized in order to carry out the statistical analysis. Inferential statistics, such as t-tests, chi-square tests, and correlation analysis, were utilized in order to evaluate the relationships between metabolic parameters and cardiovascular autonomic function. Descriptive statistics were utilized in order to provide a summary of the baseline characteristics. The results were deemed statistically significant when the p-value was less than 0.05.

## Results

The study recruited 100 individuals diagnosed with T2DM along with 100 control participants, for comparative analysis. Notably, the group with T2DM demonstrated significantly higher levels of HbA1C compared to the control group. Specifically, the average HbA1C level in the T2DM group was 7.95%, with a standard deviation of 1.17, while the control group had an average HbA1C level of 5.00% and a standard deviation of 0.56. This marked difference underscores the metabolic challenges faced by those with T2DM (Table 1).

**Table 1:** HbA1C Levels in Study Groups HbA1C: Glycosylated Hemoglobin; T2DM: Type 2 Diabetes Mellitus

Group	Sample Size	Mean HbA1C (%)	Standard Deviation
T2DM	100	7.95	1.17
Control	100	5.00	0.56

Analysis revealed a significant association between HbA1C levels and various parameters of cardiovascular autonomic function within the T2DM cohort. If there is a higher level of HbA1C, the HR would be faster at rest (mean difference = 10 bpm, p < 0.001) and the blood pressure would react differently during autonomic function tests. This included systolic blood pressure (mean difference = 15 mmHg, p < 0.01) and diastolic blood pressure (mean difference = 10 mmHg, p < 0.05). High glycemic variability was also linked to problems with controlling heart and blood vessel function (correlation coefficient = 0.75, p < 0.001) (Table 2).

**Table 2:** Association between HbA1C Levels and Cardiovascular Autonomic Function Bpm: Beats Per Minute; SBP: Systolic Blood Pressure; DBP: Diastolic Blood Pressure

Parameter	Mean Difference/Correlation Coefficient	p-value
Resting Heart Rate (bpm)	10	<0.001
SBP Response (mmHg)	15	<0.01
DBP Response (mmHg)	10	<0.05
Glycemic Variability	0.75	<0.001

There were also dyslipidemia levels in the T2DM group, with higher amounts of TGs (T2DM: mean = 200 mg/dL, SD = 40 mg/dL; Control: mean = 150 mg/dL, SD = 30 mg/dL) and LDL cholesterol (T2DM: mean = 160 mg/dL, SD = 30 mg/dL; Control: mean = 100 mg/dL, SD = 20 mg/dL) and lower amounts of HDL cholesterol (T2DM: mean = 40 mg/dL, SD = 10 mg/dL; Control: mean = 50 mg/dL, SD = 15 mg/dL) (Table 3).

**Table 3:** Lipid Profile in Study Groups LDL: Low-Density Lipoprotein; HDL: High-Density Lipoprotein

Parameter	T2DM Mean (SD)	Control Mean (SD)
Triglycerides (mg/dL)	200 (40)	150 (30)
LDL Cholesterol (mg/dL)	160 (30)	100 (20)
HDL Cholesterol (mg/dL)	40 (10)	50 (15)

The findings indicate a significant relationship between BMI and changes in cardiovascular autonomic function, as shown by a correlation coefficient of 0.60 and a highly significant statistical result (p < 0.001). This suggests that obesity is a key factor contributing to cardiovascular autonomic dysfunction in individuals with T2DM.

Moreover, the duration of diabetes was found to have a detrimental impact on cardiovascular autonomic function. There was a notable decline associated with each additional year of living with diabetes, quantified as a mean decrease of 0.5 points per year, which was statistically significant (p < 0.01). This highlights the progressive nature of the adverse effects that prolonged diabetes can have on cardiovascular health

Finally, higher amounts of inflammatory markers like CRP (mean = 5 mg/L, SD = 2 mg/L) and IL-6 (mean = 10 pg/mL, SD = 4 pg/mL) were found in the T2DM group. This suggests that they were in a pro-inflammatory state that might be causing their cardiovascular autonomic function to get worse (Table 4).

**Table 4:** Inflammatory Marker Levels in the T2DM Group CRP: C-Reactive Protein; IL-6: Interleukin 6

Marker	Mean (SD)
CRP (mg/L)	5 (2)
IL-6 (pg/mL)	10 (4)

## Discussion

In patients who were diagnosed with T2DM, the objective of this study was to investigate the relationship between metabolic regulation and the autonomic function of the cardiovascular system. The results of this study shed light on the complicated relationship between metabolic abnormalities and cardiovascular autonomic dysfunction in T2DM [17]. This has implications for figuring out who is at the highest risk and how to best help them.

Our findings demonstrated significantly higher HbA1C values in T2DM patients compared to control participants, indicating inadequate metabolic management in the diabetic population. These findings are in line with those of prior research that has highlighted the key role that glycemic control plays in the etiology of diabetic sequelae, including cardiovascular disease [18,19]. During autonomic function evaluations, it was discovered that elevated HbA1C levels were related to a higher resting HR and abnormal BP responses. This finding suggests that there may be a connection between poor glycemic management and autonomic dysfunction in T2DM [18,19].

Glycemic variability became another important factor in determining cardiovascular autonomic function in T2DM. Changes in blood glucose levels outside the normal range were linked to problems with controlling autonomic function. This finding underscores the importance of not only achieving target HbA1C levels but also maintaining stable blood glucose levels to prevent cardiovascular complications in T2DM [18,19].

Low HDL cholesterol levels are a hallmark of the condition known as dyslipidemia, which also has high levels of triglycerides and LDL cholesterol. This was prevalent in individuals with T2DM and further contributed to the impairment of cardiovascular autonomic regulation [20,21]. The link found between dyslipidemia and autonomic dysfunction shows that people with T2DM need to have their lipid levels managed along with their blood sugar levels to lower their cardiovascular risk [22,23].

Higher BMI values were shown to be independently linked with changes in cardiovascular autonomic function measures. This finding highlights the importance of being overweight as a preventable risk factor for cardiovascular problems in T2DM [24]. The prolonged duration of diabetes was also associated with greater impairment in autonomic function, suggesting a cumulative effect of metabolic dysregulation on the autonomic nervous system over time [25].

Furthermore, increased levels of inflammatory markers, including CRP and IL-6, were observed in the T2DM group, indicative of a pro-inflammatory state potentially contributing to the deterioration of cardiovascular autonomic function [26]. Targeting inflammation may represent a novel therapeutic approach for preventing cardiovascular complications in T2DM [27].

Limitations

One of the primary limitations of our study is its cross-sectional nature, which hampers our ability to ascertain causal relationships. Additionally, the recruitment of participants from a single location may limit the generalizability of our findings to a broader population. To validate our results and gain a deeper insight into the mechanisms connecting metabolic imbalances with cardiovascular autonomic disturbances in T2DM, future research should incorporate longitudinal studies with larger and more diverse participant groups.

## Conclusions

The results of our research offer knowledge that is crucial for understanding the intricate interaction that exists between metabolic management and cardiovascular autonomic function in people with T2DM. By addressing metabolic irregularities, such as glycemic control, dyslipidemia, obesity, and inflammation, it may be possible to provide possibilities for risk reduction and tailored interventions in order to prevent cardiovascular problems in this high-risk population. It is necessary to do additional research in order to better understand the mechanisms that are at play and to develop therapeutic approaches that are optimal for improving cardiovascular outcomes in people with T2DM.

## References

[1] Dilworth L, Facey A, Omoruyi F (2021). Diabetes mellitus and its metabolic complications: the role of adipose tissues. Int J Mol Sci.

[2] Hlyan NP, Arif T, Jaufar SS (2024). From sugar spikes to pressure peaks: navigating the world of diabetes, hypertension, obesity, and kidney health. Cureus.

[3] Islam R, Islam H (2023). Comment on: perceived heart attack likelihood in adults with a high diabetes risk. Heart Lung.

[4] Zakir M, Ahuja N, Surksha MA (2023). Cardiovascular complications of diabetes: from microvascular to macrovascular pathways. Cureus.

[5] Ma CX, Ma XN, Guan CH, Li YD, Mauricio D, Fu SB (2022). Cardiovascular disease in type 2 diabetes mellitus: progress toward personalized management. Cardiovasc Diabetol.

[6] Flores Monar GV, Islam H, Puttagunta SM (2022). Association between type 1 diabetes mellitus and celiac disease: autoimmune disorders with a shared genetic background. Cureus.

[7] Sudo SZ, Montagnoli TL, Rocha BS, Santos AD, de Sá MP, Zapata-Sudo G (2022). Diabetes-induced cardiac autonomic neuropathy: impact on heart function and prognosis. Biomedicines.

[8] Qasim M, Rashid MU, Islam H, Amjad D, Ehsan SB (2021). Knowledge, attitude, and practice of diabetic patients regarding foot care: experience from a single tertiary care outpatient clinic. Foot (Edinb).

[9] Yu TY, Lee MK (2021). Autonomic dysfunction, diabetes and metabolic syndrome. J Diabetes Investig.

[10] Chen J, Yin D, Dou K (2023). Intensified glycemic control by HbA1c for patients with coronary heart disease and Type 2 diabetes: a review of findings and conclusions. Cardiovasc Diabetol.

[11] Belli M, Bellia A, Sergi D, Barone L, Lauro D, Barillà F (2023). Glucose variability: a new risk factor for cardiovascular disease. Acta Diabetol.

[12] Rohm TV, Meier DT, Olefsky JM, Donath MY (2022). Inflammation in obesity, diabetes, and related disorders. Immunity.

[13] Jha SB, Rivera AP, Flores Monar GV (2022). Systemic lupus erythematosus and cardiovascular disease. Cureus.

[14] Ruze R, Liu T, Zou X (2023). Obesity and type 2 diabetes mellitus: connections in epidemiology, pathogenesis, and treatments. Front Endocrinol (Lausanne).

[15] (2023). Heart disease and diabetes. Diabetes in America [Internet].

[16] Okdahl T, Wegeberg AM, Pociot F, Brock B, Størling J, Brock C (2022). Low-grade inflammation in type 2 diabetes: a cross-sectional study from a Danish diabetes outpatient clinic. BMJ Open.

[17] Islam R, Islam H (2023). Comment on: Long-term outcomes in patients with acute myocardial infarction and no ischemic changes on electrocardiogram. Heart Lung.

[18] S Jarab A, Al-Qerem WA, Hamam H, Abu Heshmeh S, Al-Azzam S, L Mukattash T, Alefishat EA (2023). Glycemic control and its associated factors among diabetic heart failure outpatients at two major hospitals in Jordan. PLoS One.

[19] Islam H, Islam R (2023). Cardiovascular outcomes of patients referred to home based cardiac rehabilitation. Heart Lung.

[20] Huang L, Pan Y, Zhou K, Liu H, Zhong S (2023). Correlation between glycemic variability and diabetic complications: a narrative review. Int J Gen Med.

[21] Chait A, Eckel RH, Vrablik M, Zambon A (2023). Lipid-lowering in diabetes: an update. Atherosclerosis.

[22] Ormazabal V, Nair S, Elfeky O, Aguayo C, Salomon C, Zuñiga FA (2018). Association between insulin resistance and the development of cardiovascular disease. Cardiovasc Diabetol.

[23] Csige I, Ujvárosy D, Szabó Z, Lőrincz I, Paragh G, Harangi M, Somodi S (2018). The impact of obesity on the cardiovascular system. J Diabetes Res.

[24] Kumari K, Kumar R, Memon A (2023). Treatment with testosterone therapy in type 2 diabetic hypogonadal adult males: a systematic review and meta-analysis. Clin Pract.

[25] Drozdz D, Alvarez-Pitti J, Wójcik M (2021). Obesity and cardiometabolic risk factors: from childhood to adulthood. Nutrients.

[26] Stanimirovic J, Radovanovic J, Banjac K (2022). Role of C-reactive protein in diabetic inflammation. Mediators Inflamm.

[27] Goldfine AB, Shoelson SE (2017). Therapeutic approaches targeting inflammation for diabetes and associated cardiovascular risk. J Clin Invest.

